# Growth of *Mycobacterium tuberculosis* biofilms containing free mycolic acids and harbouring drug-tolerant bacteria

**DOI:** 10.1111/j.1365-2958.2008.06274.x

**Published:** 2008-05-27

**Authors:** Anil K Ojha, Anthony D Baughn, Dhinakaran Sambandan, Tsungda Hsu, Xavier Trivelli, Yann Guerardel, Anuradha Alahari, Laurent Kremer, William R Jacobs, Graham F Hatfull

**Affiliations:** 1 Department of Biological Sciences, University of PittsburghPittsburgh, PA 15260, USA; 2 Howard Hughes Medical Institute, Department of Microbiology and Immunology, Albert Einstein College of Medicine1300 Morris Park Avenue, Bronx, NY 10461, USA; 3 Unité de Glycobiologie Structurale et Fonctionnelle, CNRS UMR 8576, Université des Sciences et Technologies de Lille59650 Villeneuve d'Ascq, Cédex, France; 4 Laboratoire de Dynamique des Interactions Membranaires Normales et Pathologiques, Université de Montpellier II et I, CNRSUMR 5235, case 107, Place Eugène Bataillon, 34095 Montpellier Cedex 05, France; 5 INSERM, DIMNP, Place Eugène Bataillon34095 Montpellier Cedex 05, France

## Abstract

Successful treatment of human tuberculosis requires 6–9 months' therapy with multiple antibiotics. Incomplete clearance of tubercle bacilli frequently results in disease relapse, presumably as a result of reactivation of persistent drug-tolerant *Mycobacterium tuberculosis* cells, although the nature and location of these persisters are not known. In other pathogens, antibiotic tolerance is often associated with the formation of biofilms – organized communities of surface-attached cells – but physiologically and genetically defined *M. tuberculosis* biofilms have not been described. Here, we show that *M. tuberculosis* forms biofilms with specific environmental and genetic requirements distinct from those for planktonic growth, which contain an extracellular matrix rich in free mycolic acids, and harbour an important drug-tolerant population that persist despite exposure to high levels of antibiotics.

## Introduction

Human tuberculosis infections typically require treatment with multiple antibiotics for 6–9 months to avoid re-emergence of the disease (Hopewell *et al*., 2006). The reasons why such extended treatments are required are not completely understood, but as antibiotics effectively kill *Mycobacterium tuberculosis* during the first 14 days of treatment (Jindani *et al*., 2003; Sirgel *et al*., 2005), a subpopulation of drug-tolerant cells that are either inaccessible or non-responsive to small molecules must persist (Wallis *et al*., 1999). Current models of *M. tuberculosis* persistence typically invoke the presence of dormant populations of non-growing cells that reactivate upon host immunosuppression (Wayne and Sohaskey, 2001). While *in vitro* data indicate that active growth is important for susceptibility of *M. tuberculosis* to many front-line antibiotics (Xie *et al*., 2005), mutant strains that are defective in *in vitro* dormancy do not appear defective in *in vivo* persistence (Parish *et al*., 2003; Voskuil *et al*., 2003; Rustad *et al*., 2008).

In other infectious diseases, such as *Pseudomonas aeruginosa* in cystic fibrosis and *Escherichia coli* in urinary tract infections, biofilms provide an important reservoir of cells that can repopulate colonized sites upon removal of drug treatment (Singh *et al*., 2000; Anderson *et al*., 2004). As a correlation between biofilm formation and bacterial persistence has been proposed (Costerton *et al*., 1999; Balaban *et al*., 2004; Roberts and Stewart, 2005), the question arises as to whether *M. tuberculosis* can form drug-tolerant biofilms. If so, it raises the possibility of *M. tuberculosis* biofilm formation as a potential new target for drugs that facilitate the use of current anti-tuberculosis antibiotics administered in ultra-short regimens. In a surfactant-free liquid medium culture, *M. tuberculosis* can form organized pellicle-like structures on the air–media interface (Darzins and Fahr, 1956), although the growth characteristics and persistence of the pathogen in these multicellular communities have not been closely scrutinized.

Cellular and molecular studies on biofilms of several mycobacterial species have been performed (Schulze-Robbecke and Fischeder, 1989; Hall-Stoodley and Lappin-Scott, 1998; Marsollier *et al*., 2005; Hall-Stoodley *et al*., 2006). *Mycobacterium smegmatis* forms biofilms both on PVC surfaces and on liquid–air interfaces, and exhibits sliding motility on agar surface; the genetic requirements for biofilm formation and sliding motility are similar (Recht and Kolter, 2001; Ojha *et al*., 2005; Ojha and Hatfull, 2007). *M. smegmatis* biofilm formation requires a low level of supplemental iron that is acquired by the exochelin uptake system (Ojha and Hatfull, 2007) and is genetically distinct from planktonic growth (Recht and Kolter, 2001; Ojha *et al*., 2005). Unlike most other biofilm-forming bacteria, *M. smegmatis* biofilms contain a lipid-rich extracellular matrix and the FAS-II system responsible for the synthesis of mycolic acids is implicated in this process (Ojha *et al*., 2005). In particular, mutations in both *inhA* and *kasB* give rise to biofilm defects, and GroEL1 – a dedicated chaperone for biofilm formation – specifically interacts with components of the FAS-II system. Mycolic acid synthesis is therefore closely associated with *M. smegmatis* biofilm formation and this is of particular note as the front-line anti-tuberculosis drug isoniazid acts on the essential InhA enzyme in mycolate synthesis (Vilcheze *et al*., 2006).

Here, we investigate the conditions that support growth of *M. tuberculosis* biofilms and demonstrate that biofilm formation is genetically and physiologically distinct from planktonic growth of *M. tuberculosis*. These biofilms are unusual in that the tubercle bacilli are embedded in a lipid-rich extracellular matrix containing free methoxy mycolic acids. Moreover, we show that *M. tuberculosis* biofilms are drug-tolerant and harbour persistent cells that survive high concentrations of anti-tuberculosis antibiotics.

## Results and discussion

### Growth of *M. tuberculosis* biofilms

**Fig. 1:**
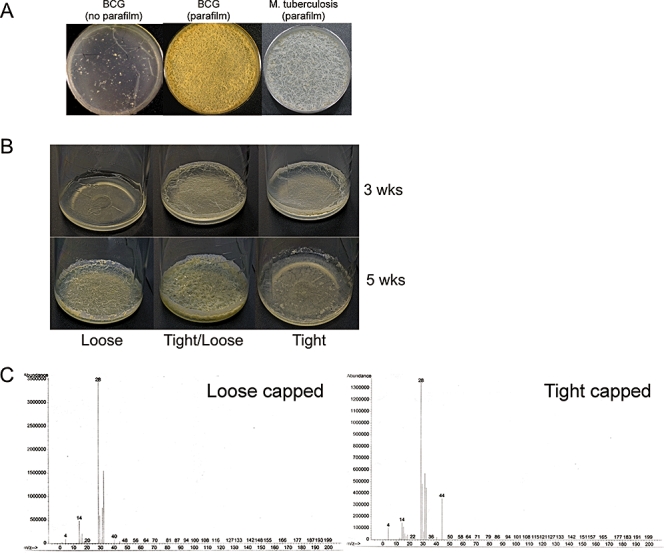
Growth of *M. tuberculosis* biofilms. *A. M. bovis* BCG or *M. tuberculosis* H37Rv was cultured in a Petri dish containing Sauton's medium for 5 weeks with either a standard loose-fitting lid or with a sealed parafilm covering. B. Bottle assay for the growth of *M. tuberculosis* mc^2^7000 biofilms. Bottle caps were kept either loose or tight for 5 weeks, or tight for the first 3 weeks and loosened for the last 2 weeks, as indicated. C. Gases present in either loosely capped or tightly capped bottles (as indicated) were examined by GC/MS. CO_2_ (44 amu) accumulates in the tightly capped bottles.

Biofilm growth of non-tuberculosis mycobacteria is dependent on growth conditions and medium*. M. tuberculosis* does not grow in the modified M63 medium that supports formation of *M. smegmatis* biofilms at liquid–air interfaces (Recht *et al*., 2000; Branda *et al*., 2005; A.K. Ojha and G.F. Hatfull, unpubl. obs.) and, in an established medium, such as Sauton's, we found that both *M. tuberculosis and Mycobacterium bovis* BCG form a thin film in a Petri dish without the thick textured reticulation that is typical of mature biofilms of *M. smegmatis* and other mycobacteria (Fig. 1A). However, serendipitously we observed that when using the same Sauton's medium, the sealing of the dishes with parafilm supports growth of lush mature biofilms by both *M. bovis* and *M. tuberculosis* (Fig. 1A) after 5 weeks of incubation, which are visually indistinguishable from those of *M. smegmatis*.

We propose that there are two aspects of these parafilm-covered dishes that are important for biofilm growth and maturation. First, as parafilm is likely to restrict the loss of gases and volatile substances by diffusion, we suggest that these volatile agents may either mediate signalling events or satisfy specific metabolic requirements needed for biofilm maturation. Second, because biofilm maturation of other mycobacteria requires continued bacterial growth, it seems likely that the property of parafilm to permit diffusion of oxygen is an important parameter. We note that this underscores a significant difference with models of mycobacterial dormancy which require a micro-anaerobic environment (Wayne and Sohaskey, 2001).

We have reproduced *M. tuberculosis* biofilm growth using polystyrene bottles, which provide more robust and reproducible conditions. In this format, the medium occupies approximately 10% of the bottle volume and is inoculated by adding cells from a saturated planktonic culture at a 1:100 dilution. If the bottle is tight-capped, then after 3 weeks of incubation, attachment of *M. tuberculosis* cells to the sides of the polystyrene bottles and some maturation is observed, but there is little change to the biofilm appearance over the subsequent 2 weeks of incubation (Fig. 1B). In contrast, if the bottle caps are kept loosely associated, then biofilm formation is noticeably retarded, and little attachment or maturation is observed after 3 weeks' incubation, and is only apparent after 5 weeks of incubation (Fig. 1B). We find that optimal biofilm formation is observed by maintaining the bottle tightly capped for 3 weeks, and then loosening the caps for the last 2 weeks (Fig. 1B). This is a simple and robust biofilm model that – even though the extent and timing of biofilm development may vary with bottles and caps – enables analysis of gas accumulation under these conditions.

To identify putative gases and volatile substances that are required for biofilm formation, we analysed the gases by gas chromatography/mass spectrometry (GC/MS), comparing those in the head-space of a tight-capped bottle used to grow *M. tuberculosis* biofilms with those in a loose-capped bottle (Fig. 1C). This analysis showed accumulation of CO_2_ (44 amu) and lower abundance of oxygen (32 amu) in the tight-capped bottle (Fig. 1C). Other volatile substances may be present, but at concentrations too low for detection by GC/MS. A critical role for CO_2_ seems implausible as we would expect it to diffuse through a parafilm cover (Fig. 1A).

### Role of carbon dioxide in *M. tuberculosis* biofilm formation

**Fig. 2:**
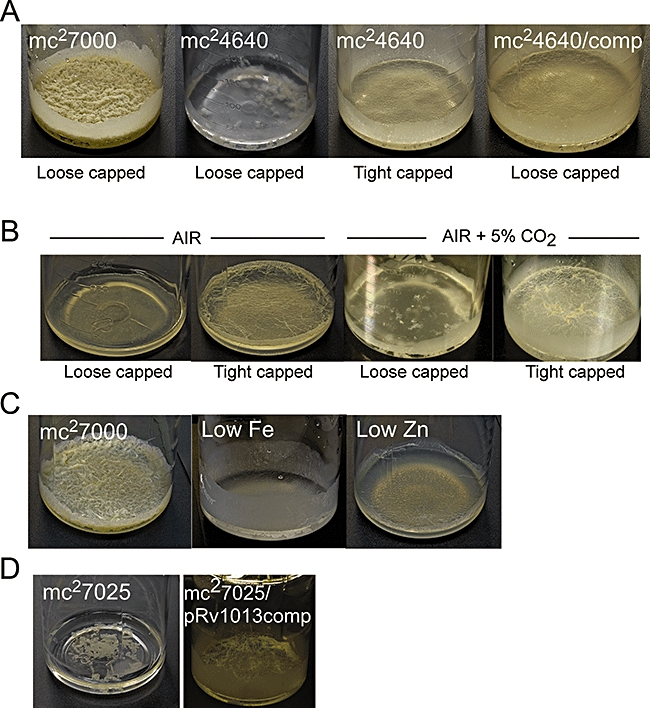
Environmental and genetic requirements for *M. tuberculosis* biofilms. A. *M. tuberculosis* mc^2^4640, a mutant deleted for genes encoding 2-oxoglutarate dehydrogenase, requires CO_2_ for colony formation on solid media and is defective in biofilm formation. Tightly capping the bottle for the first 3 weeks or complementing with genes *Rv2454c-Rv2455c* (mc^2^4640/comp) partially restores biofilm formation; all cultures were grown for 5 weeks. B. Exogenous CO_2_ (5%) is not sufficient to support full growth of *M. tuberculosis* biofilms. Biofilms were grown in air or air supplemented with 5% CO_2_ as shown. C. Low iron (2 μM) and low zinc (none added) inhibit biofilm development with no effect on planktonic growth (see Fig. S1). D. *M. tuberculosis* strain mc^2^7025 defective in gene *Rv1013* is strongly defective in biofilm formation, and complemented by plasmid pRv1013comp that contains the *Rv1013* gene.

Phenotypic analysis of mutants attenuated in the ability to produce CO_2_ suggests that CO_2_ availability plays a rolein *M. tuberculosis* biofilm formation. For example, a *M. tuberculosis* mutant (mc^2^4640) that lacks the genes for 2-oxoglutarate dehydrogenase (encoded by *Rv2454c* and *Rv2455c*; A. D. Baughn and W. R. Jacobs, unpubl. data) and requires exogenous CO_2_ for growth on solid medium is defective in biofilm formation (Fig. 2A). Biofilm formation by this strain was partially restored either by precluding gas exchange (tightening the cap of the culture vessel) during the first 3 weeks of incubation, or by complementing the genetic defect by integration of the wild-type *Rv2454c-Rv2455c*locus (Fig. 2A). We recognize that this mutant is likely to have substantial alterations in its overall carbon metabolism and, owing to the presence of other similarly prominent metabolic sources of CO_2_, namely isocitrate dehydrogenase and pyruvate dehydrogenase, it is possible that CO_2_ accessibility is not the sole factor necessary to promote *M. tuberculosis* biofilms. Yet, it is important to note that biofilm formation of the wild-type strain was stimulated by supplementation with 5% CO_2_, although to a far lesser degree than sealing of the culture vessel (Fig. 2B). Overall, these data suggest that changes in the composition of the gaseous space other than CO_2_ accumulation – such as decreasing oxygen tension or increasing concentration of organic volatile molecules – stimulate biofilm development. We have not been able to identify these by GC/MS and the nature of the stimulant remains elusive.

### Requirement of iron and zinc for *M. tuberculosis* biofilm maturation

Iron plays a central role in biofilm formation (Banin *et al*., 2005; Ojha and Hatfull, 2007) and we therefore examined the iron dependence of *M. tuberculosis* biofilms. Reducing the normal amount of iron (to 2 μM) has no effect on planktonic growth (Fig. S1) but severely retards biofilm growth (Fig. 2C). The effect is most apparent on the maturation stages, and bacteria remain competent to attach to the sides of the polystyrene bottles (Fig. 2C). The requirement of iron specifically for late biofilm development rather than attachment is similar to that reported for *M. smegmatis*, although we note that 2 μM iron is sufficient to support full *M. smegmatis* biofilm growth (Ojha and Hatfull, 2007). Removal of the zinc supplement also interrupts *M. tuberculosis* biofilm development (Fig. 2C) without affecting planktonic growth (Fig. S1); interestingly, both iron and zinc are important metal cofactors for enzymes involved in CO_2_ generation and capture as well as synthesis of mycolic acids. We note, for example, that carbonic anhydrase requires zinc for generating carbonate from CO_2_, which is utilized by carboxybiotin-dependent acetyl CoA carboxylases to make the malonyl-CoA and methylmalonyl CoA precursors for mycolic acid biosynthesis; whereas FeS cluster proteins are required for synthesis of the biotin cofactor.

### *Mycobacterium tuberculosis* biofilm growth is genetically distinct from planktonic growth

To determine if biofilm formation can be genetically differentiated from planktonic growth, we isolated mutants from a transposon insertion library of *M. tuberculosis* that have reduced biofilm growth (D. Sambandan and W. R. Jacobs Jr, unpublished); one such mutant, mc^2^7025, is strongly defective (Fig. 2D), while growing normally in planktonic culture (Fig. S1). The biofilm defect is severe and, even under optimal conditions, there is a nearly complete loss of liquid surface growth, although there is still visible attachment to the surfaces of the polystyrene bottles. The transposon insertion maps to gene *Rv1013* encoding a putative acyl-CoA synthase and, although the genes and its product are not well-characterized, it is reported to be upregulated about twofold after 24 h starvation (Betts *et al*., 2002); complementation with Rv1013 restores the biofilm phenotype (Fig. 2D). A second mutant – defective in *helY* – shows a similar biofilm defective phenotype (data not shown). Clearly, biofilm formation is a genetically distinct state from planktonic growth or growth on solid agar medium.

### *Mycobacterium tuberculosis* biofilms contain abundant extractable free mycolic acids

**Fig. 3:**
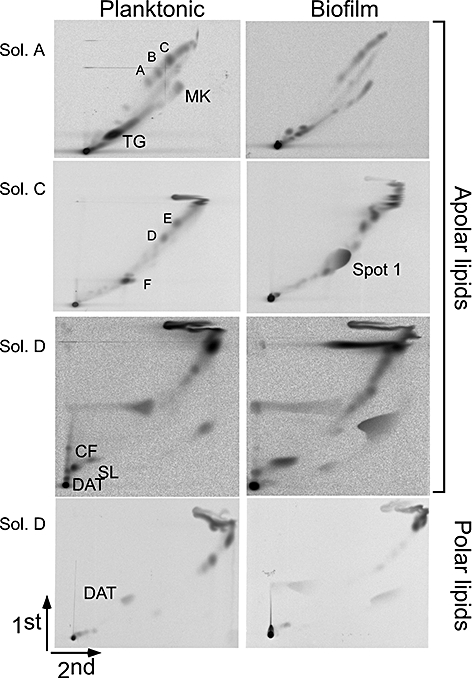
Lipid analysis of planktonically grown cells and biofilms of *M. tuberculosis*. Total apolar and polar lipids from planktonic and biofilm cultures of *M. tuberculosis* mc^2^7000 resolved on 2D-TLC are solvent systems as described in the *Experimental procedures* (solvent A, Sol. A; solvent C, Sol. C; solvent D, Sol. D). Samples were labelled with ^14^C-acetate and equal amounts of total radioactivity were examined. The lipids are annotated as previously described (Besra, 1998): A–C, phthiocerol dimycoserosate family; MK, menaquinone; D and E, apolar mycolipenates of trehalose; F, free fatty acid; CF, cord factor; DAT, 2,3,-di-O-acyltrehaloses; SL, sulphatides. Spot 1 which is induced in biofilms is also marked.

*Mycobacterium tuberculosis* biofilms are unusual in that they contain novel lipids which are most likely derivatives of mycolic acids (Ojha *et al*., 2005). Elevated level of these lipids is closely associated with the late stages of biofilm maturation and a Δ*groEL1* mutant defective in maturation fails to make these lipids (Ojha *et al*., 2005). We therefore examined whether *M. tuberculosis* biofilms contain lipids that are not present in planktonic cultures. Initially, we extracted both apolar and polar lipids from planktonic and biofilm cultures and compared them by two-dimensional thin-layer chromatography (TLC) as described previously (Dobson *et al*., 1985) (Fig. 3). Overall, the lipid profiles for the two samples are very similar, with two notable exceptions. First, triacylglyceride (TG) is present in planktonically grown cells as expected, but is present at a reduced level in the biofilm sample (Fig. 3A); presumably, TG is being used as an intracellular carbon source in biofilm growth, as has been shown for starvation conditions (Deb *et al*., 2006). Interestingly, extraction of apolar lipids and analysis by TLC with solvent C (see *Experimental procedures*) show the presence of a fatty acid (labelled Spot 1 in Fig. 3) that is much more abundant in the biofilm culture than in planktonic cells.

**Fig. 4:**
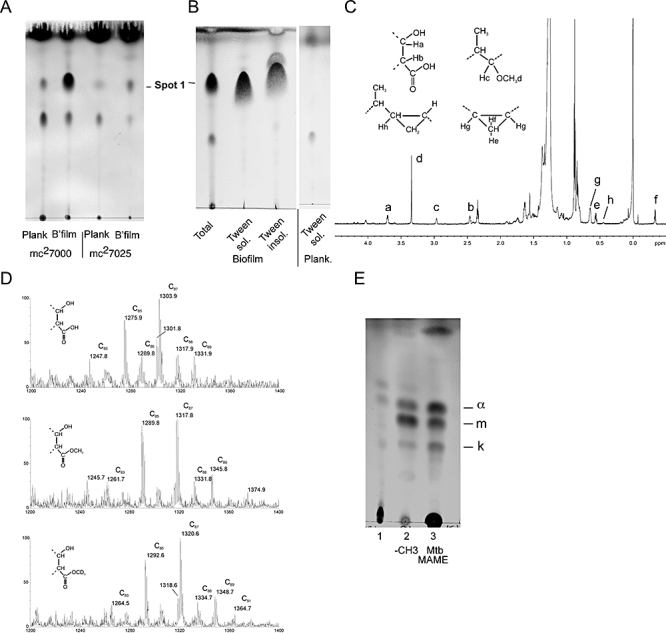
*M. tuberculosis* biofilms contain free mycolic acids. A. 1D-TLC of apolar lipids extracted from either *M. tuberculosis* mc^2^7000 cells or the mc^2^7025 mutant growing either planktonically or as biofilms resolved in solvent system C. Samples were labelled with ^14^C-acetate and equal amounts of total radioactivity were examined. B. Tween-80 solubilization of biofilm lipids. Biofilm samples were Tween-80 extracted and the Tween-soluble and Tween-insoluble fractions separated by TLC as above. The Tween-containing supernatant from planktonically grown cells was analysed similarly. C. ^1^H-NMR spectrum of Spot 1. ^1^H signals of protons associated with functional groups were assigned using known chemical shifts (Watanabe *et al*., 1999; X. Trivelli and Y. Guerardel, unpubl. obs.) and labelled (a to h) according to their relative positions. A slight de-shielding of Ha (Δδ + 0.06 p.p.m.) proton from 3.64 to 3.70 p.p.m. and Hb (Δδ + 0.03 p.p.m.) proton from 2.43 to 2.46 p.p.m. compared with their equivalent protons associated to a methyl-esterified carboxyl group is in agreement with the occurrence of a free mycolate. D. MALDI-TOF-MS analysis of native (top panel), -CH3 (middle panel) and -CD3 (bottom panel) esterified Spot 1. Signals correspond to [M + Na]^+^ adducts of a family of methoxy mycolates. E. Spot 1 (lane 1) was methyl-esterified (lane 2) and compared with methyl-esterified mycolic acids purified from *Mtb* cell wall (lane 3), by resolution on a TLC plate developed in petroleum ether : acetone (95:5) and visualized as in Fig. 4A. a, m and k indicate the alpha, methoxy and keto mycolic acids respectively.

Separation of Spot 1 by TLC and quantification shows that it is present in about fivefold greater abundance in biofilms than in planktonically grown cells (Table S1). Furthermore, the abundance of Spot 1 is reduced in mc^2^7025 mutant biofilms grown under the same conditions as the parental strain (Fig. 4A), indicating that accumulation of spot 1 closely correlates with formation of mature biofilms structures. We further tested whether Spot 1 is anchored to the mycobacterial cells within a biofilm, in which case a strong chemical hydrolysis would be required to isolate it, or if it is extracellular and can be readily extracted by a mild detergent treatment. As shown in Fig. 4B, a substantial proportion of the Spot 1 material is readily extracted with Tween-80, consistent with it being extracellular and presumably a component of the extracellular matrix. To eliminate the possibility that Spot 1 is also present in planktonically grown cells but lost from the sample as a result of solubilization with detergent, we analysed the supernatant of a planktonic culture, but found that Spot 1 is absent (Fig. 4B). Spot 1 is thus specifically made by *M. tuberculosis* growing in biofilms.

Chemical analysis of Spot 1 shows that it corresponds to free mycolic acids. First, we characterized the Spot 1 lipid by ^1^H-NMR, which shows specific signals indicative of the presence of functional groups of mycolic acids (Fig. 4C). In addition to the protons adjacent to the carboxyl group that are ubiquitously observed in mycolic acids (Fig. 4C, Ha and Hb), *cis*-cyclopropyl (Fig. 4C, He, Hf and Hg) and α-methyl methoxy groups (Fig. 4C, Hc, Hd) are readily identified on the ^1^H-NMR spectrum and confirmed by ^1^H-^13^C HSQC NMR experiments (data not shown), strongly suggesting the presence of methoxy mycolates as major components, and probably α mycolates. In addition, minor signals assigned to keto groups were observed in small amounts, establishing the presence of keto mycolates as minor compounds (about 10%). However, no signals indicative of *cis*- or *trans*-double bonds were observed, ruling out the occurrence of α_1_ and α_2_ mycolates. Most informative are the ^1^H-NMR chemical shifts of the -CHa- and -CHb- signals that are noticeably de-shielded from 3.64 to 3.70 p.p.m. and from 2.43 to 2.46 p.p.m., respectively, compared with their equivalents when the carboxyl group is methyl-esterified (Watanabe *et al*., 2001), which suggests that the carboxyl group is free. Accordingly, ^1^H-^13^C HMBC experiment of Spot 1 (data not shown) did not show any correlation between the carbon of the carboxyl group and a proton of a potential carrier molecule through a ^3^*J*_H-C_. Furthermore, although very close, the NMR parameters of Hb also slightly differ from those of the -CH_2_-COOH group of a free fatty acid (2.34 p.p.m.) because of the de-shielding effect of the hydroxyl group in the beta position of mycolic acids. This demonstrates that signals associated with free carboxyl groups do not originate from a contamination of free linear fatty acids in Spot 1. These NMR data strongly suggest that the carboxyl group of the mycolic acid is not engaged in linkage with the C5 carbon of an arabinosyl residue or the C6 carbon of a glucose residue – as observed in mAGP or in TDM respectively – but is free.

The MALDI-TOF analysis of Spot 1 (Fig. 4D) shows several major signals between m/z 1247 and 1331 with 14 mu increments fitting with [M + Na]^+^ adducts either of C_82_-C_86_ methyl-esterified methoxy-mycolic acids (Fujita *et al*., 2005) or, in accordance with the NMR data, of a family of C_83_-C_87_ free methoxy mycolates. In agreement with this hypothesis, esterification of Spot 1 with methyl groups (-CH_3_) in aqueous strong alkali solution led to a mass increment of 14 mu of all the signals, demonstrating the presence of a methylable function, presumably the free carboxylic group, on the molecule. Values of methylated compounds from m/z 1261–1345 fit with those of C_83_-C_87_ methyl-esterified methoxy mycolates, as previously described (Fujita *et al*., 2005). It is noteworthy that the respective intensities of each signal before and after methylation are almost identical (Fig. 4D), strongly suggesting that both families of compounds are indeed related. In addition, esterification of Spot 1 with deuteromethyl (-CD_3_) group incremented all signals by 17 mu, which unambiguously demonstrated that the 14 mu increment was the result of the addition of a single methyl group to the mycolic acid. Finally, ^1^H-NMR analysis of the methylated product shows an almost identical spectrum to that of the native molecule, only differing by the chemical shifts of the -CHa- and -CHb- groups that shift back to their typical values for methyl-esterified mycolic acids, demonstrating that methylation occurred on the free carboxyl group. This was further confirmed by ^1^H-^13^C HMBC analysis of methylated Spot 1 (data not shown) that showed a clear ^3^*J*_H-C_ correlation between the carbon of the carboxyl group and the proton of the methyl group. TLC analysis of methyl-esterified Spot 1 also confirmed the presence of methoxy mycolates with lesser amounts of both α and keto mycolates (Fig. 4E). Thus taken together, NMR, MS and TLC analyses of native and methyl-esterified Spot 1 unambiguously confirm that it is composed of free mycolic acids, an observation of some interest as we are not aware of any other condition in which free mycolic acids are abundant components of *M. tuberculosis* cultures.

Re-examination of the biofilm-associated fatty acids of *M. smegmatis* reported previously (Ojha *et al*., 2005) suggests that these are also free mycolic acids (data not shown). Apolar lipids with similar properties to those in *M. tuberculosis* can be extracted without hydrolysis and are at reduced levels in the Δ*groEL1* mutant (data not shown). Thus, the development of biofilm-associated extracellular matrices composed of free mycolic acids is not unique to *M. tuberculosis*, and it seems more likely that these are common features of mycobacterial biofilms. Furthermore, the production of these *M. smegmatis* lipids does not appear to be via *de novo* synthesis (Ojha *et al*., 2005), but rather a GroEL1-mediated switch in the action of the FAS-II system. Because the mycolates are usually covalently attached to the cell surface, it is plausible that the free mycolic acids primarily are generated by cleavage of the ester linkages anchoring them to arabinogalactan, although a putative esterase that could be responsible for this has yet to be identified. This suggests the possibility that mycolic acids play two separate but important roles in mycobacterial physiology: conferring cohesion and structure to biofilm communities of cells, and only being cell-associated when mycobacterial cells are free to migrate from one community to another. We also note that while a *groEL1* mutant of *M. tuberculosis* exhibits no obvious biofilm phenotype under the standard conditions for biofilm growth described here (A.K. Ojha and G.F. Hatfull, unpubl. obs.), *M. tuberculosis groEL1* is required for granulomatous inflammation in both mice and guinea pigs (Hu *et al*., 2008).

### *Mycobacterium tuberculosis* biofilms contain highly drug-tolerant persister cells

**Fig. 5:**
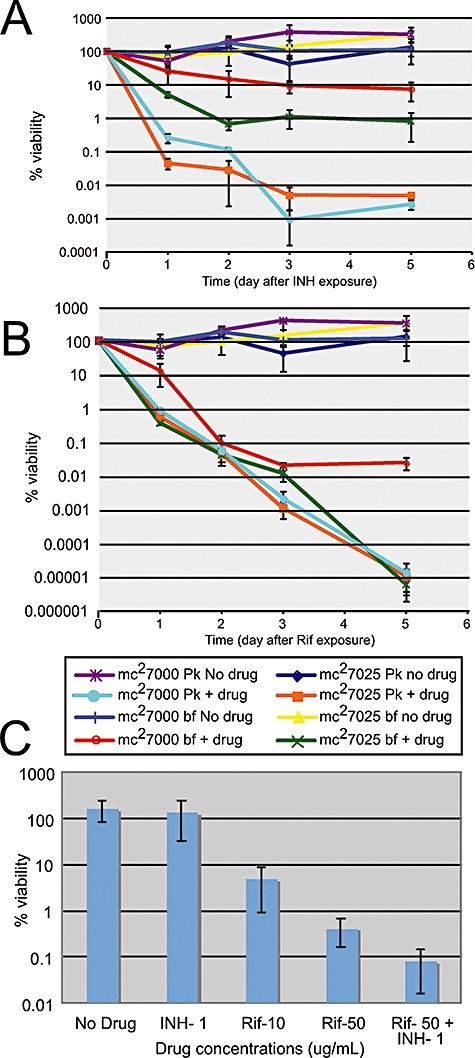
*M. tuberculosis* biofilms are drug-tolerant. *M. tuberculosis* mc^2^7000 or the biofilm-defective mc^2^7025 mutant were grown planktonically or as biofilms and treated with either isoniazid (A) or rifampicin (B); the percentages of viable cells remaining after different times of drug treatment are shown. (C) Percentage of surviving cells following 7 day treatment of *M. tuberculosis* biofilms with antibiotics at different concentrations.

Bacterial biofilms typically have a high proportion of cells that are non-responsive to antimicrobials, and these likely correspond to persisters that arise as a result of phenotypic variation in a subpopulation of heterogeneous bacilli and are seen in a variety of microbial species (Spoering and Lewis, 2001; Dhar and McKinney, 2007; Lewis, 2007). We therefore tested whether *M. tuberculosis* biofilms also display phenotypic variation in response to antibiotics (Fig. 5). In planktonic growth, incubation with either isoniazid or rifampicin leads to cell death and the rate of killing is linear over a 5 day period. In contrast, *M. tuberculosis* biofilms have a high proportion of cells (∼10%) that survive isoniazid treatment, even at concentrations (10 μg ml^−1^) greatly above the minimal inhibitory concentration (MIC) (∼0.1 μg ml^−1^). As isoniazid fails to kill non-growing cells, this may simply mean that 10% of the cells are not in an active growing state, although we note that the mc^2^7025 mutant is nearly 10-fold more sensitive than its parent strain when grown under these conditions (Fig. 5A).

In contrast, *M. tuberculosis* biofilms are fully responsive to high rifampicin exposure over a 3 day period with a > 1000-fold reduction in viability (Fig. 5B); however, further exposure causes no reduction in viability, indicating a substantial subpopulation of cells surviving this treatment. At lower concentrations of rifampicin (10 μg ml^−1^) – still substantially above the MIC (∼1 μg ml^−1^) – an even greater proportion of drug-tolerant persisters was observed (∼8%; Fig. 5C) following 7 days of exposure. Reducing the INH concentration to 1 μg ml^−1^ somewhat reduced the proportion of drug-tolerant biofilm cells, but did exhibit a modest synergistic effect when used in combination with rifampicin (Fig. 5C).

This biphasic killing curve in response to rifampicin treatment (Fig. 5A and B) is characteristic of cultures that contain a substantial proportion of persisters, cells that are not killed by the antibiotics but remain genetically sensitive (Dhar and McKinney, 2007); in patients, *M. tuberculosis* is killed rapidly over the first 2 days of drug treatment, but more slowly thereafter (Jindani *et al*., 2003). We have analysed the colonies recovered after 5 days of rifampicin treatment, and all tested remain fully antibiotic-sensitive. *M. tuberculosis* mc^2^7025 does not contain these persisters (Fig. 5B), demonstrating that drug tolerance is biofilm-dependent.

## Conclusions

In this study, we have shown that *M. tuberculosis* forms pellicular biofilms that are distinct from planktonic growth. We have demonstrated that the *M. tuberculosis* requires a specific environment and genetic programme to form organized biofilm communities which harbour drug-tolerant cells within a structure rich in free mycolic acids. Although the question as to whether *M. tuberculosis* forms biofilms during infectious growth has received little attention, within the primary granulomas in guinea pig lungs, an acellular rim has been observed adjacent to the edge of the mineralizing central necrosis containing drug-tolerant bacteria in microcolonies with features reminiscent of biofilms (Lenaerts *et al*., 2007). Taken together with our demonstration that *M. tuberculosis* forms biofilms as a defined and distinct physiological state harbouring drug-tolerant persisters, we propose that biofilm development represents an excellent drug target. Identification of drugs that inhibit biofilm formation could enable the dramatic shortening of tuberculosis treatments using standard antibiotics, with substantial potential impact on global health and reduction of antibiotic resistance associated with non-compliance.

## Experimental procedures

### Bacterial strains, media and growth conditions

*Mycobacterium tuberculosis* mc^2^7000 is an unmarked version of mc^2^6030 (Sambandamurthy *et al*., 2006) and the *hyg sacB* cassette was removed by transduction with phAE87 containing the γδ resolvase and screening for sucrose-resistant and hygromycin-sensitive isolates. mc^2^7000 fails to kill SCID mice following intravenous challenge and is ultimately cleared and has been approved for use in Biosafety Level 2 containment by the Institutional Biosafety Committees of the Albert Einstein College of Medicine and University of Pittsburgh. Unless otherwise stated, *M. bovis* BCG (Pasteur) and *M. tuberculosis* mc^2^7000 were grown planktonically in 7H9OADC with 0.05% Tween-80 in 5% CO_2_ and mc^2^7000 was supplemented with 100 μg ml^−1^ of pantothenic acid. Antibiotics hygromycin (75 μg ml^−1^) and kanamycin (25 μg ml^−1^) were added as appropriate.

### Biofilm growth

Biofilms of *M. bovis* BCG and *M. tuberculosis* (mc^2^7000) were grown in polystyrene Petri dishes by inoculating 10 ml of Sauton's medium (without Tween-80) with 100 μl of a saturated planktonic culture and incubating without shaking at 37°C for 5 weeks in humidified conditions. The dishes were either unwrapped or wrapped with parafilm during incubation. Biofilms of *M. tuberculosis* strains (mc^2^7000, mc^2^4640, mc^2^4640-comp, mc^2^7025) were also grown in 250 ml polystyrene bottles by inoculating 25 ml of Sauton's media with 250 μl of a saturated planktonic culture and incubating without shaking at 37°C for 5 weeks. Bottles were incubated either loose- or tight-capped in air or with 5% CO_2_.

### Isolation of *M. tuberculosis* biofilm-defective mutants

A TnHimar transposon library of *M. tuberculosis*, mc^2^7000 (Bardarov *et al*., 1997), was screened for mutants defective in the formation of biofilms in 96-well plates. Single colonies were inoculated into Sauton's media without Tween-80 and incubated at 37°C for 5 weeks (D. Sambandan *et. al.*, in preparation). Transposon insertion sites were determined by junction cloning and DNA sequencing. Construction of *M. tuberculosis* mc^2^4640 was constructed by replacing *Rv2454c* and *Rv2455c* for 2-oxoglutarate dehydrogenase (nucleotides 2753746–2756555 corresponding to the published H37Rv genome sequence, Cole *et al*., 1998) of mc^2^7000 with a hygromycin-resistance cassette (Bardarov *et al*., 2002); mc^2^4640-comp was constructed by transformation with a pYUB1052 derivative (Braunstein *et al*., 2000) containing the *Rv2454c-Rv2455c* region (H37Rv nucleotides 2721932–2757635).

### GC-MS analysis

Biofilms of mc^2^7000 were grown in 24 ml of Sauton's media in 236 ml screw-capped glass bottles (with hole caps and septa) in either tightened or loosened-lid conditions. After 3 weeks of incubation, the lids of all the bottles were tightened and the air from the overhead space withdrawn and injected into a GC-MS instrument (Hewlett Packard) with Agilent J and W column. The instrument was maintained at 20°C for the entire run time and the mass spectra of the GC peaks were collected between 1 and 200 amu.

### Drug tolerance assays

For the drug tolerance assay, biofilms of *M. tuberculosis* (mc^2^7000 and mc^2^7025) were grown in 12-well polystyrene tissue culture plates, each well containing 6 ml of Sauton's media and inoculated with 60 μl of saturated planktonic culture. The plates were parafilm-wrapped and incubated at 37°C for 5 weeks. One set of cultures was harvested at time t_0_ by collecting the biomass in 0.2% Tween-80, gentle agitation at 4°C for 24 h with 1 mm glass beads and plated for cell viability. From the remaining wells, one set was kept as ‘no-drug control’ and to the rest, either isoniazid (20 μg ml^−1^) or rifampicin (50 μg ml^−1^) was injected through the biofilm into the media. Cells were harvested at various times with 0.2% Tween-80, washed twice with Sauton's medium, re-suspended in 6 ml of the same medium, gently agitated at 4°C for 24 h with 0.1% Tween and 1 mm glass beads, and cell viabilities determined. Planktonic cultures of the mc^2^7000 and mc^2^7025 at 0.2 OD were exposed to the same drug concentrations and viabilities determined.

### Lipid extraction and analysis

*Mycobacterium tuberculosis* was grown planktonically to OD 0.2 or in biofilms for 5 weeks, labelled with 1.0 μCi ml^−1^ of ^14^C acetate for 24 h prior, and harvested. Cells were re-suspended in 5 ml of methanol: 0.3% NaCl (100:10), mixed with 2.5 ml of petroleum ether for 30 min at room temperature, and the upper petroleum ether layer containing the apolar lipids separated by centrifugation. After solvent evaporation, apolar lipids were dissolved in 0.5 ml of Dichloromethane, and amounts equivalent to 50 000 counts of each sample were analysed by silica two-dimensional TLC developed in solvent systems A (1st dimension – petroleum ether/ethyl acetate 98:2 three times, 2nd dimension – petroleum ether/acetone 98:2), C (1st dimension – chloroform/methanol 96:4, 2nd dimension – toulene/acetone 80:20) and D (1st dimension – chloroform/methanol/water 100:14:0.8, 2nd dimension – chloroform/acetone/methanol/water 50:60:2.5:3) described previously (Dobson *et al*., 1985). Lipids equivalent to 50 000 c.p.m. were loaded from each sample and visualized by spraying 5% molybdophosphoric acid.

The polar lipids from the menthanol-saline bottom layer were extracted following the same protocol and the lipids were resolved on 2D-TLC using solvent system D.

Free and cell-associated lipids were separated by extraction of 5 week biofilms in 15 ml of PBS containing 0.2% Tween-80 and 1 mm glass beads for 24 h, followed by centrifugation at 6000 r.p.m. for 15 min. Apolar lipids were extracted from the supernatant and the cell pellet as described above. For further analysis of Spot 1, the lipid was scraped off a TLC plate, extracted thrice in diethyl ether and dried.

### Mass spectrometry

Mass spectrometry analyses of Spot 1 were done on a Voyager Elite reflectron MALDI-TOF mass spectrometer (PerSeptive Biosystems, Framingham, MA, USA), equipped with a 337 nm UV laser. Samples were solubilized in 1 ml of chloroform/methanol (2:1) of which 1 μl was mixed with 1 μl of 2,5-dihydroxybenzoic acid matrix solution (10 mg ml^−1^ dissolved in chloroform/methanol 2:1). Spot 1 was analysed in native and esterified forms; dried samples were treated in 1 ml of 15% tetrabutyl ammonium hydroxide at 100°C overnight, esterified by adding 2 ml of chloroform, 1 ml of water and 150 ml of either ICH3 or ICD3 and sonicated for 1 h. Methyl-esterified samples were extracted twice with chloroform/water mixture and dried under a stream of nitrogen.

### NMR analyses

Samples were dissolved into deuterated chloroform containing 0.03% of TMS for ^1^H and ^13^C referring and transferred into Shigemi tubes matched for D_2_O. 0.1 ml of deuterium oxide was added to avoid solvent evaporation during long acquisition (Wieruszeski *et al*., 2006). 1D proton, 2D-COSY, 2D-ROESY with 400 and 450 ms mixing time, 2D-^13^C-HSQC with multiplicity editing during selecting step and 2D-HMBC experiments were recorded at 299K on a 800 MHz Avance II and 400 MHz Avance Bruker spectrometers at 299K equipped with a ^1^H/^13^C/^15^N/^2^H and a broad-band probes respectively.

## Supplementary material

This material is available as part of the online article from: http://www.blackwell-synergy.com/doi/abs/10.1111/

j.1365-2958.2008.06274.x

(This link will take you to the article abstract).
