# Supplementary material for: Growth of Mycobacterium tuberculosis biofilms containing free mycolic acids and harbouring drug-tolerant bacteria
Source: Mol Microbiol. 2008 May 27;69(1):164–74. doi: 10.1111/j.1365-2958.2008.06274.x (PMC2615189; doi:10.1111/j.1365-2958.2008.06274.x)
Supplement: Supplementary file 1 [file mmi0069-0164-SD1.pdf]

Supplementary Data

Figure S1. A. Planktonic growth of *M. tuberculosis mc^{2}7000* in normal Sauton's media, modified Sauton's media with 2μM ferric ammonium citrate and modified Sauton's media without any zinc supplement.

B. Comparison of planktonic growth of mc^{2}7000 and mc^{2}7025 in Sauton's media.

Table S1. Densitometric analysis of spot1 and spot2 from Fig 4A.

Sample	Spot 1 density	Spot 2 density	Ratio (Spot1/Spot2)
mc^{2}7000- planktonic	3.83	7.05	0.54
mc^{2}7000- biofilms	18.53	5.25	3.53
mc^{2}7025- planktonic	1.24	3.21	0.39
mc^{2}7025 - biofilms	3.55	1.20	2.96

Spot 1 is as indicated in Fig. 4A. The slowest migrating lipid in Fig. 4A is spot 2. Samples for loading were normalized by total radioactivity.

A

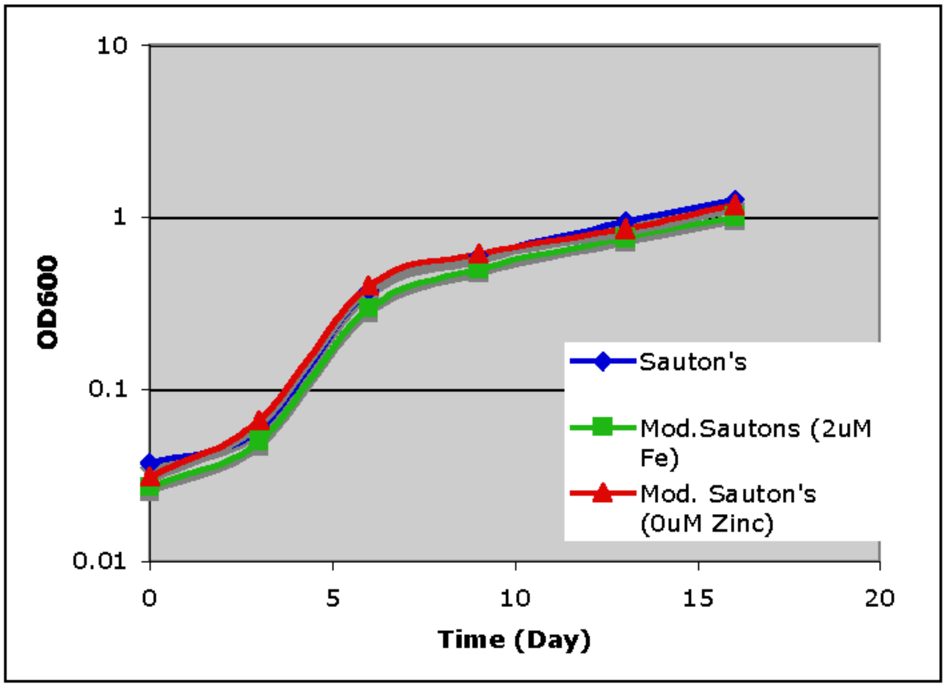
10
1
0.1
0.01
OD600
0
5
10
15
20
Time (Day)
Sauton's
Mod.Sautons (2uM Fe)
Mod. Sauton's (0uM Zinc)

B

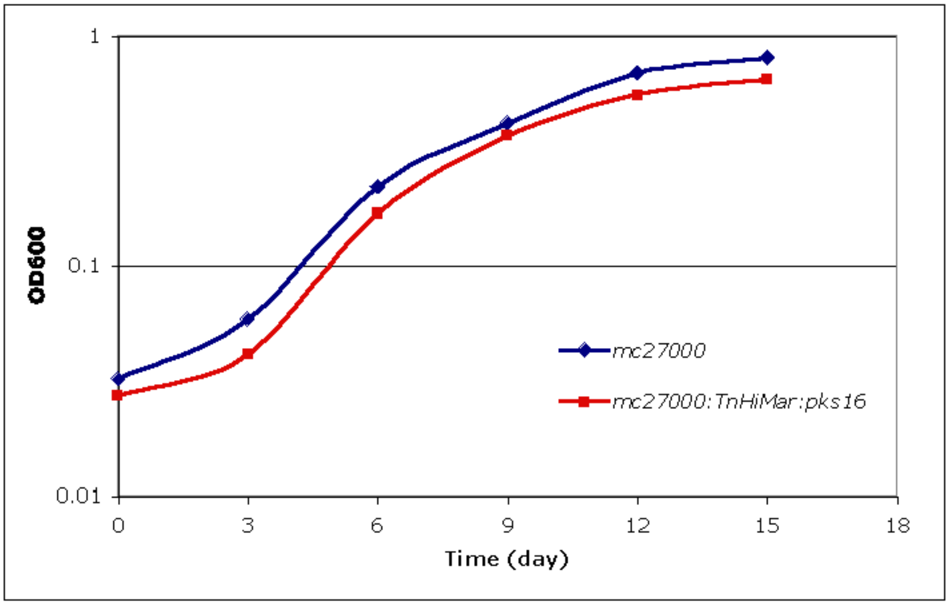
1
0.1
0.01
OD600
0
3
6
9
12
15
18
Time (day)
mc27000
mc27000:TnHiMar:pks16

Figure S1
